# *Mycobacterium riyadhense* Pulmonary Disease after Relocation from Saudi Arabia, Japan

**DOI:** 10.3201/eid3203.251418

**Published:** 2026-03

**Authors:** Takuya Ozawa, Takeshi Komine, Sohei Nakayama, Yusuke Suzuki, Naoki Hasegawa, Koichi Fukunaga, Ho Namkoong, Hanako Fukano, Takanori Asakura

**Affiliations:** Keio University School of Medicine, Tokyo, Japan (T. Ozawa, N. Hasegawa, K. Fukunaga, H. Namkoong, T. Asakura); Japan Institute for Health Security, Tokyo (T. Komine, H. Fukano); Kitasato University Kitasato Institute Hospital, Tokyo (S. Nakayama, Y. Suzuki, T. Asakura); Kitasato University School of Pharmacy, Tokyo (Y. Suzuki, T. Asakura)

**Keywords:** *Mycobacterium* *riyadhense*, nontuberculous mycobacteria, NTM, tuberculosis and other mycobacteria, bacteria, respiratory infections, pulmonary disease, endogenous progression, case report, Japan

## Abstract

We report a case of *Mycobacterium riyadhense* pulmonary disease in a patient who relocated from Saudi Arabia to Japan. Epidemiologic data and whole-genome analyses of the isolated strains suggested that the infection might have been acquired in Saudi Arabia and persisted, rather than a recent local acquisition in Japan.

*Mycobacterium riyadhense*, first isolated in Saudi Arabia, has been reported mainly in the Middle East (*1*) and sporadically elsewhere (*2*,*3*). We describe a patient who experienced slowly progressive pulmonary deterioration caused by *M. riyadhense* infection after she relocated from Saudi Arabia to Japan. Because *M. riyadhense* has not been reported in Japan, genomic analysis of the patient’s isolates was more consistent with within-host persistence of a preexisting infection than recent local acquisition from environmental exposure in Japan.

A 47-year-old woman was referred to Kitasato University Kitasato Institute Hospital (Tokyo, Japan) after granular opacities were detected in the right lung on screening. She had lived in Saudi Arabia for 2 years, where she had chronic exposure to sand and dust. A visibly contaminated, uncleaned air-conditioning unit at her home housed a bird’s nest for 7 months and remained in use. She took only showers and rarely cleaned the shower room. She also gardened regularly. Shortly before her initial visit for care, she returned to Japan, bringing back only clothing and no other household belongings. She resumed tub bathing; the showerhead was replaced 4 years after her return, while her illness was being monitored. 

Computed tomography (CT) revealed multiple small nodular opacities in the right upper and middle lobes and the lingular segment, along with bronchial wall thickening; those findings suggested the nodular bronchiectatic form of nontuberculous mycobacterial pulmonary disease (Figure 1, panel A, B). Bronchial wash from the right upper lobe was negative for acid-fast bacilli (AFB) by smear and culture. Because she was asymptomatic, we monitored her for 2 years. CT imaging showed progressive worsening (Figure 1, panel C, D). A repeat bronchial wash from the same site in the right upper lobe was negative by AFB smear; culture yielded *M. riyadhense*, identified by matrix-assisted laser desorption/ionization time-of-flight (MALDI-TOF) mass spectrometry using the MALDI Biotyper system with the Mycobacteria Library version 6.0 (Bruker, https://www.bruker.com) (*4*). Because she was asymptomatic without lung cavities, we deferred treatment.

**Figure 1:**
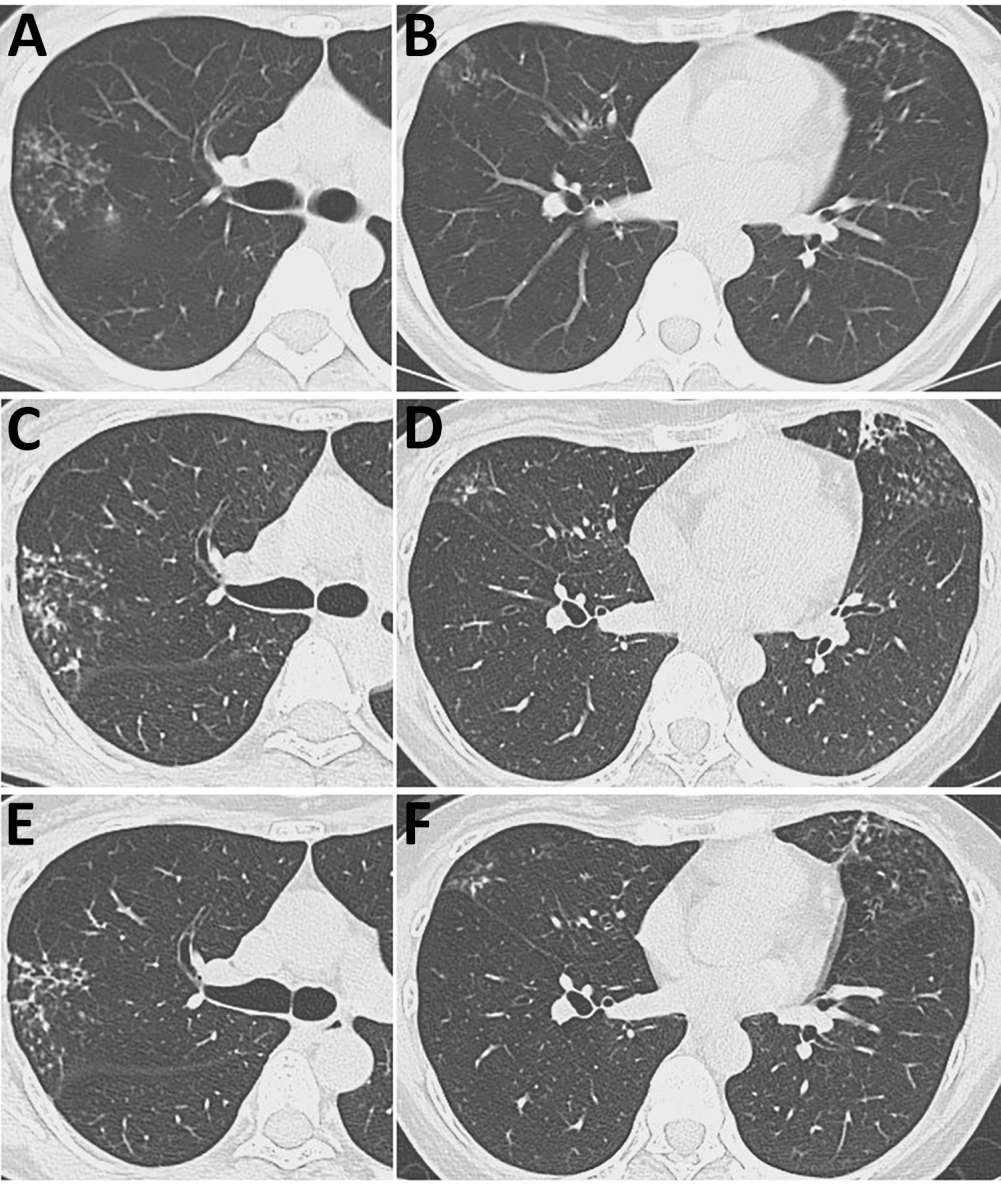
Serial axial chest computed tomography images over time from patient with *Mycobacterium riyadhense* pulmonary disease after relocation from Saudi Arabia, Japan. A, B) Images taken at initial hospital visit, demonstrating multiple scattered small nodular opacities in the right upper and middle lobes (A) and the lingular segment (B), accompanied by bronchial wall thickening. C, D) Images taken 2 years later, showing progression of the lesions in the right upper/middle lobes (C) and lingular segment (D). E, F) Images taken after treatment showing improvement of the lesions in the right upper/middle lobes (E) and lingular segment (F).

Five years after her initial visit, radiology-detected progression prompted a third bronchoscopy. Bronchial washes from 2 sites yielded *M. riyadhense* (strains 484719 and 537489), which we confirmed by MALDI-TOF mass spectrometry. We assembled draft genomes of the 2 strains from Illumina MiniSeq short-read sequencing data (https://www.illumina.com) using SPAdes version 3.15.5 (https://github.com/ablab/spades) (Appendix 1). Average nucleotide identity heatmap analysis using PyANI version 0.2.12 (https://github.com/widdowquinn/pyani) demonstrated that the isolates clustered with *M. riyadhense*, with >99.08% identity (Appendix 1 Figure 1; Appendix 2 Table 1). Phylogenetic analysis based on 4,753 core genes from 12 *M. riyadhense* genomes, including publicly available genomes from the National Center for Biotechnology Information database (Appendix), further showed that isolates from both specimens were closely related to strains reported from Saudi Arabia (Figure 2). We called 7 single-nucleotide polymorphisms (SNPs) using Snippy version 4.6.0 (https://github.com/tseemann/snippy) and Gubbins version 3.4 (https://github.com/nickjcroucher/gubbins) within the 2 isolated strains (Appendix 1 Figures 2, 3). Fourteen-day broth microdilution susceptibility testing showed favorable results (Appendix 2 Table 2). Four months later, sputum culture also yielded *M. riyadhense*. Azithromycin (250 mg/d) plus ethambutol (500 mg/d) achieved sputum culture conversion and radiologic improvement (Figure 1, panel E, F). Sputum cultures have remained negative on repeated follow-up.

**Figure 2:**
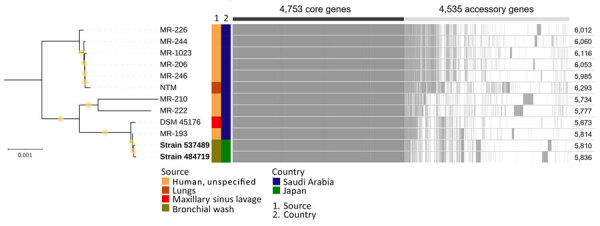
Midpoint-rooted maximum-likelihood tree based on 4,753 core genes of *Mycobacterium riyadhense* isolates from study of *Mycobacterium riyadhense* pulmonary disease after relocation from Saudi Arabia to Japan. Strains 484719 and 537489 (bold), obtained from clinical specimens in this study, were more closely related to strains MR-193 and DSM 45176 from Saudi Arabia. Yellow circles indicate ultrafast bootstrap values of 100%. Numbers at right indicate the number of coding sequences detected. Scale bar represents 0.001 substitutions per site.

We did not identify published case reports of *M. riyadhense* in Japan (Appendix). Recent studies showed that shower aerosols and certain soil types are common sources of NTM exposure (*5*,*6*). The patient had prolonged exposure to such environmental conditions while living in Saudi Arabia. Although the environmental reservoir of *M. riyadhense* is not completely defined, culture-independent surveys have detected *M. riyadhense*–consistent signatures in freshwater and soil samples, which suggests those habitats could represent potential sources of exposure (*7*,*8*). Our isolates differed from MR-193 by 11–12 SNPs, whereas they were substantially more distant from other publicly available genomes. However, neither a molecular clock nor SNP threshold for *M. riyadhense* has been established, so interpretation is limited; more genomes from the same cluster are needed to infer transmission. Nevertheless, considering the patient’s exposure history, clinical course, and the absence of previous detection reports of *M. riyadhense* in Japan, we considered within-host persistence of a preexisting infection to be a plausible explanation in this case.

No standard regimen for *M. riyadhense* infection has been established. Therapeutic approaches in previous cases have varied (*1*). A study summarizing previous cases of *M. riyadhense* (*9*) demonstrated efficacy of macrolide-based regimens combined with rifampin or fluoroquinolone, reporting a cure or improvement rate of 87.5%. In the case we describe, the isolate was susceptible to macrolides and other major drugs; therefore, we selected combination therapy with azithromycin and ethambutol. After initiating therapy, sputum cultures converted to negative within 2 months, with no evidence of recurrence. Subsequent imaging confirmed improvement in the lungs, providing further support for the efficacy of macrolide-based therapy against *M. riyadhense*.

Our findings contribute to understanding of the epidemiology and clinical course of *M. riyadhense* pulmonary disease. Given our whole-genome sequencing results and the absence of previous reports in Japan, this case might represent within-host persistence of a preexisting infection, distinct from recent local acquisition from environmental sources.

Appendix 1 Additional information about a case of *Mycobacterium riyadhense* pulmonary disease after relocation from Saudi Arabia to Japan.

Appendix 2 Data from case of *Mycobacterium riyadhense* pulmonary disease after relocation from Saudi Arabia to Japan.
