# Supplementary material for: Mycobacterium riyadhense Pulmonary Disease after Relocation from Saudi Arabia, Japan
Source: Emerg Infect Dis. 2026 Mar;32(3):462–5. doi: 10.3201/eid3203.251418 (PMC13016003; doi:10.3201/eid3203.251418)
Supplement: Appendix 1 — Additional information about a case of Mycobacterium riyadhense pulmonary disease after relocation from Saudi Arabia to Japan. [file 25-1418-Techapp-s1.pdf]

Article DOI: https://doi.org/10.3201/eid3203.251418

EID cannot ensure accessibility for supplementary materials supplied by authors. Readers who have difficulty accessing supplementary content should contact the authors for assistance.

Mycobacterium riyadhense Pulmonary Disease after Relocation from Saudi Arabia, Japan

Appendix

Short-read sequencing. Two isolates (Strain 484719 and Strain 537489) were cultured in Middlebrook 7H9 supplemented with 10% oleic albumin dextrose catalase (OADC) at 37°C for 8 weeks. DNA was extracted from pellets collected via centrifugation using a previously described method (*1*). Sequencing libraries were prepared using the Nextera XT DNA library preparation kit (Illumina, USA) and paired-end sequenced on the Illumina NovaSeq X Plus platform (2 × 150 bp). The quality of the raw reads was assessed using FastQC v0.11.9 (*2*), and all analyses were performed with default settings unless stated otherwise. The sequence reads were trimmed for quality using fastp v0.23.4 (*3*). Genome sequences were de novo assembled with fastp-trimmed reads using SPAdes v3.15.5 (*4*). Completeness and contamination of the assembled genome sequences were assessed with CheckM2 v1.0.1 (*5*) and used in the following analyses. Furthermore, the genomes were annotated with the DNA Data Bank of Japan (DDBJ) Fast Annotation and Submission Tool (DFAST) (https://dfast.ddbj.nig.ac.jp) and deposited to the DDBJ/ENA/GenBank databases under the accession numbers, BAAIHH010000001-BAAIHH010000704 and BAAIHI010000001-BAAIHI010000636, respectively. The metadata for sequencing analysis are shown in Appendix 2 Table 3.

Average nucleotide identity (ANI) analysis was conducted to identify the species of isolates. Genomes of ten *M. riyadhense* strains and 17 other mycobacterial species were retrieved from the NCBI database using datasets v18.5.1 (*6*) (Appendix 2 Table 4). The assemblies were assessed with CheckM2 v1.0.1. ANI values were then calculated using FastANI v1.33 (*7*) (Appendix 2 Table 1, https://wwwnc.cdc.gov/EID/article/32/3/25-1418-App2.xlsx). Heatmap

generation and clustering were conducted using PyANI v0.2.12 with the option -m ANIb (*8*) (Appendix Figure 1).

Pan-genome analysis. In twelve *M. riyadhense* strains, including Strain 484719 and Strain 537489, pan-genome analysis was performed. The genome sequences were re-annotated using Prokka v1.14.6 (*9*), and core genes were extracted using Roary v3.13.0, with options -i 95 and -cd 99 (*10*). Based on the 4,753 core-gene alignment trimmed using trimAl v1.5.rev0 with an option -automated1 (*11*), a maximum likelihood tree was constructed using the best-fitted nucleotide substitution model (GTR+F+I) in IQ-TREE 2.4.0 (*12*). Ultrabootstrap support values were calculated with 1,000 replications. The generated phylogenies were visualized with Interactive Tree of Life (iTOL) (*13*).

Single-nucleotide polymorphism (SNP) distances. Paired-end short-read sequencing data of eight *M. riyadhense* strains were downloaded with SRA-tool kit v3.0.3 (https://github.com/ncbi/sra-tools) from the NCBI Sequence Read Archive. Whole-genome alignment was generated with fastp-trimmed reads using Snippy v4.6.0, with the *M. riyadhense* DSM 45176 genome sequence (OY970456.1) as the reference. A recombination-free alignment was then generated using Gubbins v3.4 (*14*). SNP distances were calculated using snp-dists v0.8.2 (https://github.com/tseemann/snp-dists) (Appendix 1 Figure 2). Furthermore, SNP distances were also calculated for the four strains identified as more closely related in the pan-genome analysis (Appendix 1 Figure 3).

Literature search for *M. riyadhense* pulmonary disease in Japan. A comprehensive literature search was conducted using PubMed, Google Scholar, and the Japan Centra Revuo Medicina (Ichushi) databases. The objective was to identify titles and abstracts of articles published in English or Japanese up to September 2025 that reported cases of *M. riyadhense* pulmonary disease in Japan. The search strategy included relevant Medical Subject Headings (MeSH) and keywords such as "*Mycobacterium riyadhense*," "*M. riyadhense*," "Japan," and "Japanese." Additionally, the authors (T.O. and T.A.) manually reviewed reference lists of relevant publications and conference proceedings.

References

1. Komine T, Fukano H, Inohana M, Hoshino Y, Kurata O, Wada S. Draft genome sequences of 25 *Mycobacterium marinum* strains isolated from animals and environmental components in aquaria and an aquaculture farm. Microbiol Resour Announc, 2022;11:e0085122.

2. Babraham Bioinformatics. FastQC. [cited 2025 Aug 19]. https://www.bioinformatics.babraham.ac.uk/projects/fastqc

3. Chen S, Zhou Y, Chen Y, Gu J. fastp: an ultra-fast all-in-one FASTQ preprocessor. Bioinformatics. 2018;34:i884–90. PubMed https://doi.org/10.1093/bioinformatics/bty560

4. Prjibelski A, Antipov D, Meleshko D, Lapidus A, Korobeynikov A. Using SPAdes de novo assembler. Curr Protoc Bioinformatics. 2020;70:e102. PubMed https://doi.org/10.1002/cpbi.102

5. Chklovski A, Parks DH, Woodcroft BJ, Tyson GW. CheckM2: a rapid, scalable and accurate tool for assessing microbial genome quality using machine learning. Nat Methods. 2023;20:1203–12. PubMed https://doi.org/10.1038/s41592-023-01940-w

6. Sayers EW, Beck J, Brister JR, Bolton EE, Canese K, Comeau DC, et al. Database resources of the National Center for Biotechnology Information. Nucleic Acids Res. 2020;48(D1):D9–16. PubMed https://doi.org/10.1093/nar/gkz899

7. Jain C, Rodriguez-R LM, Phillippy AM, Konstantinidis KT, Aluru S. High throughput ANI analysis of 90K prokaryotic genomes reveals clear species boundaries. Nat Commun. 2018;9:5114. PubMed https://doi.org/10.1038/s41467-018-07641-9

8. Pritchard L, Glover RH, Humphris S, Elphinstone JG, Toth IK. Genomics and taxonomy in diagnostics for food security: soft-rotting enterobacterial plant pathogens. Anal Methods. 2016;8:12–24. https://doi.org/10.1039/C5AY02550H

9. Seemann T. Prokka: rapid prokaryotic genome annotation. Bioinformatics. 2014;30:2068–9. PubMed https://doi.org/10.1093/bioinformatics/btu153

10. Page AJ, Cummins CA, Hunt M, Wong VK, Reuter S, Holden MTG, et al. Roary: rapid large-scale prokaryote pan genome analysis. Bioinformatics. 2015;31:3691–3. PubMed https://doi.org/10.1093/bioinformatics/btv421

11. Capella-Gutiérrez S, Silla-Martínez JM, Gabaldón T. trimAl: a tool for automated alignment trimming in large-scale phylogenetic analyses. Bioinformatics. 2009;25:1972–3. PubMed https://doi.org/10.1093/bioinformatics/btp348

12. Minh BQ, Schmidt HA, Chernomor O, Schrempf D, Woodhams MD, Von Haeseler A, et al. IQ-TREE 2: new models and efficient methods for phylogenetic inference in the genomic era. Mol Biol Evol. 2020;37:2461. https://doi.org/10.1093/molbev/msaa131

13. Letunic I, Bork P. Interactive Tree of Life (iTOL) v6: recent updates to the phylogenetic tree display and annotation tool. Nucleic Acids Res. 2024;52(W1):W78–82. PubMed https://doi.org/10.1093/nar/gkae268

14. Croucher NJ, Page AJ, Connor TR, Delaney AJ, Keane JA, Bentley SD, et al. Rapid phylogenetic analysis of large samples of recombinant bacterial whole genome sequences using Gubbins. Nucleic Acids Res. 2015;43:e15. PubMed https://doi.org/10.1093/nar/gku1196

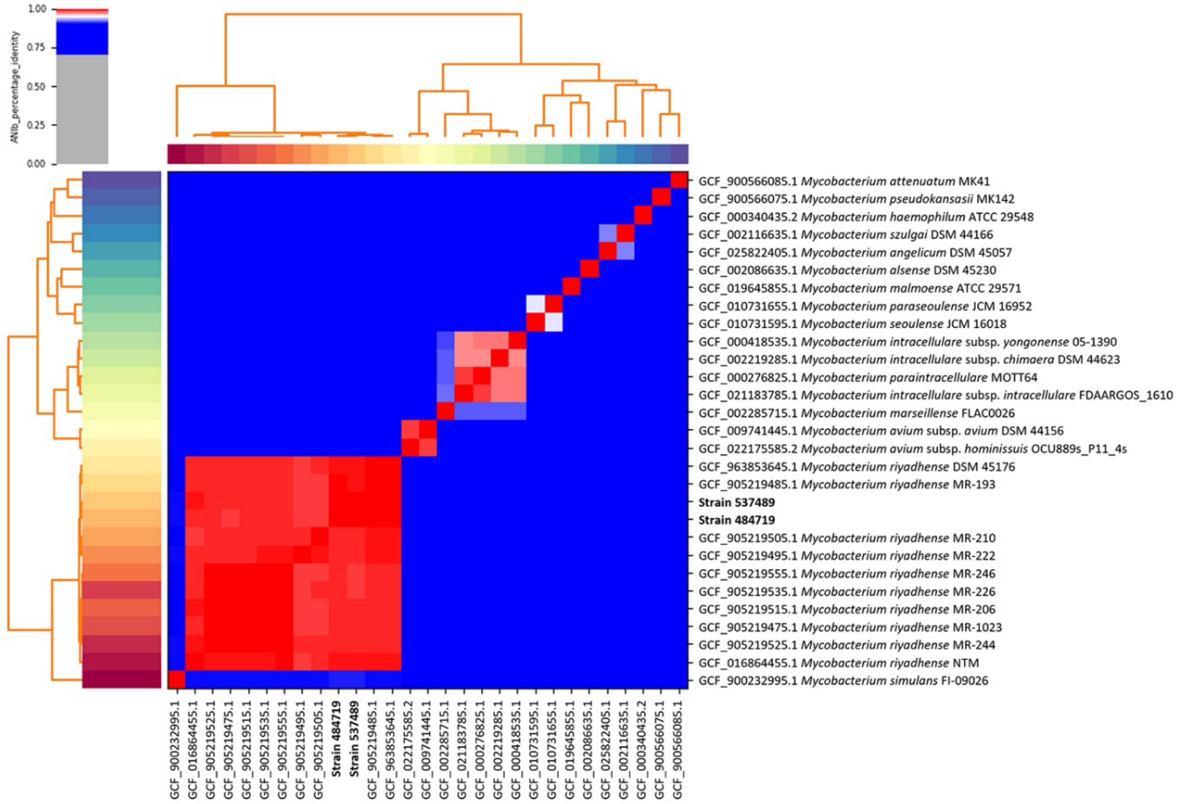

Appendix 1 Figure 1. Average nucleotide identity (ANI) heatmap of *Mycobacterium* strains. The heatmap shows pairwise ANI values among *Mycobacterium* strains. ANI values were calculated and visualized to identify the species of isolates obtained in this study (Strain 537489 and Strain 484719). The two isolates (shown in bold) clustered tightly with other *M. riyadhense* strains, demonstrating high genomic similarity consistent with classification as the same species. ANI values are color-coded, with red indicating higher identity and blue indicating lower identity. Dendrograms represent hierarchical clustering based on ANI distances.

	Strain 484719	Strain 537489	MR-193	DSM 45176	MR-222	MR-210	MR-244	MR-226	MR-246	MR-1023	MR-206
Strain 484719	0	7	11	110	10,597	11,016	11,152	11,285	11,308	11,394	11,427
Strain 537489	7	0	12	113	10,677	11,085	11,222	11,368	11,392	11,480	11,515
MR-193	11	12	0	125	11,263	11,698	11,785	12,147	12,151	12,211	12,289
DSM 45176	110	113	125	0	11,314	11,725	11,811	12,199	12,199	12,258	12,372
MR-222	10,597	10,677	11,263	11,314	0	10,861	12,038	12,313	12,338	12,342	12,342
MR-210	11,016	11,085	11,698	11,725	10,861	0	11,855	12,094	12,121	12,156	12,129
MR-244	11,152	11,222	11,785	11,811	12,038	11,855	0	223	210	246	195
MR-226	11,285	11,368	12,147	12,199	12,313	12,094	223	0	291	313	290
MR-246	11,308	11,392	12,151	12,199	12,338	12,121	210	291	0	195	133
MR-1023	11,394	11,480	12,211	12,258	12,342	12,156	246	313	195	0	182
MR-206	11,427	11,515	12,289	12,372	12,342	12,129	195	290	133	182	0

Appendix 1 Figure 2. Single nucleotide polymorphism distances within *Mycobacterium riyadhense.*

	Strain 484719	Strain 537489	MR-193	DSM 45176
Strain 484719	0	7	11	110
Strain 537489	7	0	12	115
MR-193	11	12	0	124
DSM 45176	113	115	124	0

Appendix 1 Figure 3. Single nucleotide polymorphism distances within more closely related strains of *Mycobacterium riyadhense.*
